# Association between fat mass by bioelectrical impedance analysis and bone mass by quantitative ultrasound in relation to grip strength and serum 25-hydroxyvitamin D in postmenopausal Japanese women: the Unzen study

**DOI:** 10.1186/s40101-022-00281-5

**Published:** 2022-03-09

**Authors:** Satoshi Mizukami, Kazuhiko Arima, Yasuyo Abe, Yoshihito Tomita, Hiroki Nakashima, Yuzo Honda, Michiko Uchiyama, Tetsuji Ookawachi, Hisashi Goto, Maiko Hasegawa, Youko Sou, Mitsuo Kanagae, Makoto Osaki, Kiyoshi Aoyagi

**Affiliations:** 1 Department of Public Health, Nagasaki University Graduate School of Biomedical Sciences, 1-12-4 Sakamoto, Nagasaki, 852-8523 Japan; 2 Leading Medical Research Core Unit, Nagasaki University Graduate School of Biomedical Sciences, Nagasaki, Japan; 3 Department of Health and Nutrition Science, Nishikyusyu University, Kanzaki, Japan; 4 School of Rehabilitation, Department of Physical Therapy, Tokyo Professional University of Health Science, Tokyo, Japan; 5 Ken-Hoku Health Care Office, Nagasaki, Japan; 6 Medical Policy Division, Nagasaki Prefectural Government, Nagasaki, Japan; 7 National Health Insurance & Health Improvement Division, Nagasaki Prefectural Government, Nagasaki, Japan; 8 Department of Rehabilitation, Nishi-Isahaya Hospital, Isahaya, Japan; 9 Department of Orthopedic Surgery, Nagasaki University Graduate School of Biomedical Sciences, Nagasaki, Japan

**Keywords:** Body composition, Fat mass, Muscle mass, Bone mass, Stiffness index, Postmenopausal women

## Abstract

**Background:**

Whether fat mass or lean mass affects bone mass in postmenopausal women is controversial. This study aimed to explore the association between body composition measured by bioelectrical impedance analysis (BIA) and bone mass measured by quantitative ultrasound (QUS) in postmenopausal women in Japan.

**Methods:**

We conducted a cross-sectional study, The Unzen Study, on 382 community-dwelling postmenopausal Japanese women (mean (standard deviation) age: 68.2 (7.2) years) who participated in periodic health examinations. The stiffness index (SI) was measured using QUS, and body composition (e.g., fat mass and muscle mass) was measured using BIA. Grip strength was measured. Fasting blood samples were collected, and 25-hydroxyvitamin D (25(OH)D), tartrate-resistant acid phosphatase-5b (TRACP-5b), and parathyroid hormone (PTH) levels were measured. Data on current smoking, alcohol consumption, exercise, and any comorbidities (heart disease, lung disease, stroke, or diabetes mellitus) were collected.

**Results:**

The SI increased with increasing quartiles of fat mass and muscle mass (both p for trend <  0.001), respectively. There were positive correlations between SI and log (25(OH)D) or grip strength. Fat mass significantly correlated with grip strength. Multiple linear regression analysis showed that higher fat mass was independently and significantly associated with higher SI after adjusting for age, height, comorbidity, current smoking, alcohol consumption, exercise, log (25(OH)D), log (TRACP-5b), log (PTH), and grip strength (*p* = 0.001). In contrast, no association was observed between muscle mass and SI.

**Conclusions:**

Fat mass, but not muscle mass, was a significant determinant of SI in community-dwelling postmenopausal Japanese women.

## Background

Osteoporotic patients aged 40 years and over in Japan were estimated to be approximately 12,800,000 (3,000,000 men and 9,800,000 women) based on a survey of osteoporosis in the general population [1]. Osteoporosis with bone loss is a major public health problem [2]. Age-related bone loss is associated with a fracture risk [3], high medical costs [4], and increased mortality risk [5].

Taaffe et al. [6] reported that lean mass determined using dual X-ray absorptiometry (DXA) was a significant independent contributor to bone mineral density (BMD) across race and sex. Some studies have shown that lean mass has a greater effect than fat mass on BMD in postmenopausal women after adjusting for potential confounders [7–9]. In contrast, other studies showed that BMD was related to fat mass but not to lean mass in postmenopausal women [10–12]. These results suggested biological interactions between bone mass, lean mass, and fat mass; thus, whether fat mass or lean mass affects BMD is controversial.

The gold standard for BMD measurement is DXA. However, DXA involves radiation exposure, is expensive, and has limited availability. Trimpou et al. [13] showed that the stiffness index (SI) by quantitative ultrasound (QUS) was positively correlated with BMD by DXA in each region (total, femoral neck, and lumbar spine). QUS has been shown to be a useful tool for osteoporosis screening [14–16].

Bioelectrical impedance analysis (BIA) is a method used to analyze body composition. Thomson et al. [17] reported a significant correlation between DXA and BIA for estimating fat mass and fat-free mass. Since BIA can examine a large number of individuals in a shorter period of time at a low cost, it has been used in large cohort studies, such as the National Health and Nutrition Examination Survey, USA [18].

Previous studies showed that BMD was associated with several factors, such as grip strength [19], serum 25-hydroxyvitamin D (25(OH)D) [20], serum tartrate-resistant acid phosphatase-5b (TRACP-5b) [21], and serum parathyroid hormone (PTH) [22]. Several studies reported the association between body composition and grip strength. Lean mass was significantly associated with grip strength [23]. Fat mass was positively associated [24], negatively associated [25], or not associated [26].

Previous studies have reported a relationship between body composition and BMD using DXA [6–12]. However, to the best of our knowledge, no study has examined the relationship between body composition measured by BIA and bone mass measured by QUS in postmenopausal women. DXA differentiates three components, such as fat mass, lean mass, and bone mass. On the other hand, BIA divides body composition into fat mass and fat-free mass. Muscle mass is calculated by subtracting the estimated bone mass from the fat-free mass using a BIA device (TANITA DC-320) and is similar to the lean mass measured by DXA. This study aimed to explore the association between body composition measured by BIA and bone mass measured by QUS in relation to grip strength, serum 25(OH)D, serum TRACP-5b, and serum PTH in postmenopausal women in Japan.

## Methods

### Participants

The participants were community-dwelling postmenopausal women in Unzen City, Nagasaki Prefecture, Japan, who were invited to participate in periodic health examinations between 2011 and 2013 (the Unzen study). Postmenopausal status was defined as menopause at least 1 year after the last menstruation. A total of 382 women (mean (standard deviation) age: 68.2 (7.2) years) participated in the study. All participants provided written informed consent before the examination. This study was approved by the Ethics Committee of the Nagasaki University Graduate School of Biomedical Sciences (project registration number 11072739-2). All procedures were performed in accordance with the ethical standards of the institutional and/or national research committee and in accordance with the 1964 Helsinki Declaration and its later amendments or comparable ethical standards.

### QUS measurement

The SI determined by QUS was measured on the right heel using a Lunar Achilles device (Achilles InSight GE Lunar Corp., Madison, WI, USA). It was calculated from broadband ultrasound attenuation (BUA) and speed of sound (SOS); the formula is *SI* = (0.67 × *BUA*) + (0.28 × *SOS*) – 420 [27].

### Body composition

Body composition (fat mass, percent fat, fat-free mass, and muscle mass) was measured using a TANITA DC-320 foot-to-foot body composition analyzer (TANITA Cooperation, Tokyo, Japan). A BIA system was used in this device. The participants were barefoot, wore minimal clothing, and were instructed to stand still with their feet touching all four metal plates. The percent fat was estimated using the built-in TANITA equations. Fat mass was calculated as percent fat divided by 100 and then multiplied by body weight, and fat-free mass was subsequently calculated as the difference between body weight and fat mass. The muscle mass was calculated by subtracting the estimated bone mass from the fat-free mass.

### Physical examination

Height (m) and weight (kg) were measured while wearing light clothing and without shoes, and body mass index (BMI) was calculated as weight/height squared (kg/m^2^). Grip strength was evaluated as an index of muscle strength in the upper limbs and was measured using a hydraulic hand dynamometer (Jamar hydraulic hand dynamometer; Lafayette Instrument Company, Inc., Lafayette, IN, US). Grip strength was measured twice and their excellent values were analyzed. Trained interviewers obtained information regarding the clinical characteristics of the participants. Comorbidity data, including heart disease, lung disease, stroke, and diabetes mellitus, were also collected. Data on current smoking (yes/no), alcohol consumption (≥ 20 g/day), and exercise (at least 30 min twice per week) were collected through interviews.

### Blood data

Fasting blood samples were collected. Serum 25(OH)D was measured by chemiluminescence enzyme immunoassay (CLEIA), serum TRACP-5b by enzyme immunoassay, and PTH by electrochemiluminescence immunoassay (ECLIA).

### Statistical analyses

Since 25(OH)D, TRACP-5b, and PTH were not normally distributed, these values were treated as log (25(OH)D), log (TRACP-5b), and log (PTH). Participants were classified into quartiles of fat mass and muscle mass, ranging from Q1 to Q4. Trends in SI among fat mass groups (FMQ1, FMQ2, FMQ3, and FMQ4) and muscle mass groups (MMQ1, MMQ2, MMQ3, and MMQ4) were evaluated using linear regression analysis. We applied Pearson’s product-moment correlations to assess for correlation among SI, fat mass, muscle mass, log (25(OH)D), log (TRACP-5b), log (PTH), and grip strength. Multiple linear regression analysis was used to explore the effects of fat mass and muscle mass on SI, with adjustment for age, height, comorbidity, current smoking, alcohol consumption, and exercise (Model 1) [11, 28, 29]. We added log (25(OH)D), log (TRACP-5b), log (PTH), and grip strength to model 1 (model 2) [30–32]. Statistical significance was set at *p*  < 0 .05. All statistical analyses were performed using SPSS software version 27 (SPSS Inc., Chicago, IL, USA).

## Results

Table 1 shows the characteristics of the participants. The mean (standard deviation) of age, BMI, SI, fat mass, muscle mass, and grip strength were 68.2 (7.2) years, 22.1 (3.1) kg/m^2^, 67.9 (12.7), 15.8 (5.3) kg, 33.0 (3.2) kg, and 24.2 (5.2) kg, respectively. The median of serum 25(OH)D, TRACP-5b, and PTH were 19.6ng/mL, 480.0 mU/dL, and 38.0 pg/mL, respectively. Fifteen percent of the participants had at least one comorbidity, 1.6% were alcohol drinkers, and 1.3% were current smokers. Approximately one third of the patients exercised.

**Table 1:** Characteristics of participants (*n* = 382)

Women	Total (*n* = 382)	Range
	Mean ± SD	
Age (years)	68.2 ± 7.2	49–89
Height (cm)	151.2 ± 5.6	128.5–166.6
Weight (kg)	50.6 ± 7.9	30.1–81.6
Body mass index (kg/m^2^)	22.1 ± 3.1	14.9–34.4
Stiffness index	67.9 ± 12.7	41.3–132.0
Percent fat (%)	30.5 ± 6.1	8.0–45.1
Fat mass (kg)	15.8 ± 5.3	3.1–36.9
Fat-free mass (kg)	34.9 ± 3.6	22.4–53.7
Muscle mass (kg)	33.0 ± 3.2	21.6–50.2
Grip strength (kg)	24.2 ± 5.2	6.0–39.0
Serum	Median (Q1–Q3)	
25(OH)D (ng/mL)	19.6 (15.9–23.9)	7.3–42.6
TRACP-5b (mU/dL)	480.0 (391.0–615.3)	134.0–1151.0
PTH (pg/mL)	38.0 (30.0–50.0)	10.0–159.0
	*n* (%)	
Comorbidities*	55 (14.4)	
Alcohol consumption	6 (1.6)	
Current smoking	5 (1.3)	
Exercise	140 (36.6)	

*SD* standard deviation, *25(OH)D* 25-hydroxyvitamin D, *TRACP-5b* tartrate-resistant acid phosphatase-5b, *PTH* parathyroid hormone

Data are shown as mean ± standard deviation, median (first quartile–third quartile), or n (%).

*Comorbidities included at least one of the following: heart disease, lung disease, stroke, or diabetes mellitus.

Table 2 shows the trends of SI among fat mass and muscle mass groups classified by quartile. The SI increased with increasing fat mass and muscle mass quartiles (both p for trend < 0 .001), respectively.

**Table 2:** Mean (SD) of stiffness index according to fat mass and muscle mass quartile

Variables	Q1	Q2	Q3	Q4	*p* for trend
Fat mass	(*n* = 91)	(*n* = 97)	(*n* = 98)	(*n* = 96)	
	62.7 (10.9)	68.0 (11.5)	70.2 (13.6)	70.3 (13.1)	< 0.001
Muscle mass	(*n* = 94)	(*n* = 95)	(*n* = 97)	(*n* = 96)	
	65.2 (12.6)	66.1 (11.5)	69.7 (12.8)	70.4 (13.2)	< 0.001

*SD* standard deviation

**Fig. 1:**
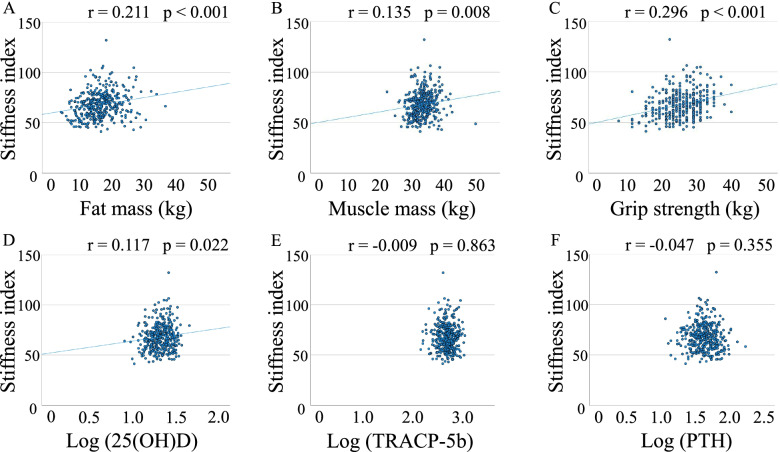
Correlations between stiffness index and fat mass (**A**), muscle mass (**B**), grip strength (**C**), log (25(OH)D) (**D**), log (TRACP-5b) (**E**), or log (PTH) (**F**)

**Fig. 2:**
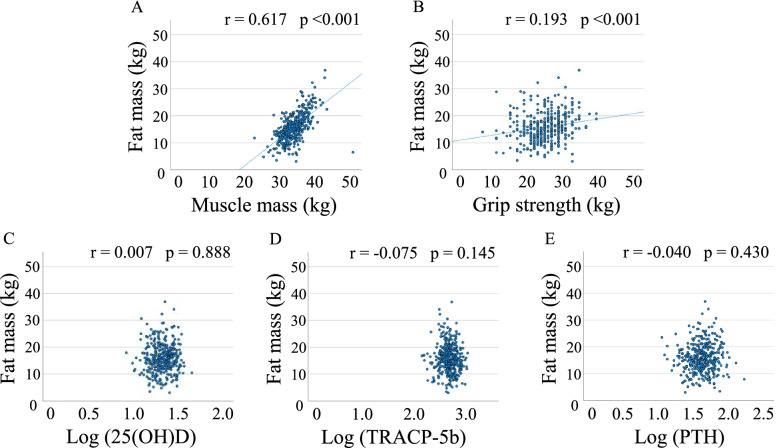
Correlations between fat mass and muscle mass (**A**), grip strength (**B**), log (25(OH)D) (**C**), log (TRACP-5b) (**D**), or log (PTH) (**E**)

There were positive correlations between SI and fat mass, muscle mass, log (25(OH)D), or grip strength, but not log (TRACP-5b) and log (PTH) (Fig. 1). Fat mass significantly correlated with muscle mass or grip strength, but not log (25(OH)D), log (TRACP-5b), or log (PTH) (Fig. 2).

Table 3 shows the results of the multiple linear regression analysis assessing the factors associated with SI in postmenopausal women. Higher fat mass was independently and significantly associated with higher SI after adjusting for age, height, comorbidities, alcohol consumption, current smoking, and exercise (*p* < 0.001) (Model 1). After adding log (25(OH)D), log (TRACP-5b), log (PTH), and grip strength to model 1, the results did not alter (Model 2). Younger age, alcohol consumption, exercise, higher log (25(OH)D), and stronger grip strength were also associated with a higher SI. In contrast, no association was observed between muscle mass and SI.

**Table 3:** Association between body composition (fat mass and muscle mass) and stiffness index

Variables	Model 1			Model 2		
	*B*	*SE*	*p* value	*B*	*SE*	*p* value
Fat mass (kg)	0.505	0.152	< 0.001	0.493	0.151	0.001
Muscle mass (kg)	− 0.374	0.307	0.223	− 0.568	0.310	0.068
Adjusted factors						
Age (years)	− 0.492	0.096	< 0.001	− 0.376	0.097	< 0.001
Height (cm)	0.160	0.156	0.306	0.086	0.156	0.579
Comorbidities*	− 1.952	1.734	0.261	− 1.004	1.712	0.558
Alcohol consumption	− 8.751	4.928	0.077	− 11.758	4.856	0.016
Current smoking	− 4.817	5.418	0.375	− 4.428	5.316	0.405
Exercise	3.772	1.263	0.003	4.133	1.242	< 0.001
log (25(OH)D)				10.180	5.025	0.043
log (TRACP-5b)				− 0.761	3.852	0.844
log (PTH)				1.253	3.537	0.723
Grip strength (kg)				0.562	0.134	< 0.001

*SE* standard error, *25(OH)D* 25-hydroxyvitamin D, *TRACP-5b* tartrate-resistant acid phosphatase-5b, *PTH* parathyroid hormone

*Comorbidities included at least one of the following: heart disease, lung disease, stroke, or diabetes mellitus

## Discussion

We examined the association of body composition (fat mass and muscle mass) measured by BIA with SI measured by QUS in Japanese postmenopausal women. Fat mass was significantly associated with SI after adjusting for covariates, whereas muscle mass was not. Several studies have shown that fat mass is the most significant predictor of BMD in postmenopausal women, compared with lean mass [6, 11, 12, 33, 34], which was consistent with our results. These findings suggest that fat mass may have a positive effect on bone formation in postmenopausal women.

Besides energy storage, fat tissues produce estrogen [35, 36]. Estrogen maintains bone mass by decreasing the lifespan of osteoclasts and increasing osteoblast and osteocyte survival [37]. The conversion of androstenedione to estrone occurs predominantly in fat tissue [38]. Fat tissues are considered the major source of circulating estrogen, and the contribution of fat tissues to the total circulating estrogens increases with advancing age [39]. Estrogen secreted by the fat mass may have a positive and significant effect on bone metabolism in postmenopausal women.

Thomas et al. [40] demonstrated the relationship of fat mass and leptin to BMD, both of which were positively correlated with BMD, and the strength of the association between fat mass and BMD decreased after adjusted serum leptin. Leptin significantly increased osteoprotegerin mRNA steady-state levels and protein secretion [41], suggesting an effect of leptin on inhibiting bone resorption. Leptin may mediate at least part of the protective effect of fat mass on bone.

Increased weight has a positive effect on BMD [42, 43] and reduces fracture risk in the elderly [10]. Osteoblasts produce new bone tissue when the daily mechanical load exceeds normal physiological levels, and osteoclasts remove existing bone tissue when the daily mechanical load falls below normal levels [44]. Previous reports suggested that microgravity contributes to bone loss [45, 46]. Goodship et al. [47] found that BMD was reduced by up to 7% in os clacis, which received no mechanical stimulus during long-term space flight. This means that bone mass and bone strength are maintained by appropriate weight-bearing. Therefore, higher weight, including higher fat mass, may contribute to good bone health.

We did not observe an association between muscle mass (corresponding to lean mass measured using DXA) and SI. Reid et al. [33] showed that lean mass was not an independent correlation of BMD at any site once fat mass was considered, which was consistent with our results. In contrast, some studies reported that lean mass is a significant determinant of BMD [6, 48]. Marin et al. [9] showed that lean mass was significantly related to all BMD sites (e.g., lumbar spine, femoral neck, and total body), whereas fat mass was only weakly related to the BMD of the femoral neck. Further research is needed to clarify whether fat and muscle mass are independent predictors of bone mass.

Log (25(OH)D) positively correlated with SI in this study. Previous studies suggested that higher serum 25(OH)D concentrations are associated with increased BMD among postmenopausal women [20, 49]. Vitamin D might be important for good bone health in postmenopausal women.

The present study showed that grip strength was positively associated with SI. Previous study demonstrated a relationship between grip strength and a higher SI [28]. These results suggest that muscle strength, but not muscle mass, is important for the maintenance of bone mass in postmenopausal women.

In this study, fat mass positively correlated with grip strength. Sherk et al. [24] reported that fat mass was significantly correlated with upper muscle strength in postmenopausal women. On the other hand, a previous study reported that fat mass of the upper limbs was negatively associated with grip strength in the elderly [25]. Moreover, Ibeneme et al. [26] demonstrated that there was no significant correlation between fat mass and grip strength in postmenopausal women. Thus, there is a controversial relationship between fat mass and muscle strength (grip strength). Further studies are needed to elucidate the association between fat mass and muscle strength (grip strength).

This study had several limitations. First, because body composition was measured using the BIA method, this study cannot be compared directly with a previous study that measured body composition using DXA. Second, because this study was conducted in a cross-sectional setting, the results do not show a causal relationship. Longitudinal studies are required to establish the causal relationships between body composition and bone mass. Third, the participants in this study were recruited from community-dwelling residents who voluntarily attended a health examination, contributing to selection bias. Fourth, the present results were obtained only from Japanese postmenopausal women; therefore, it is not possible to extrapolate the results to other ethnicities, premenopausal women, or men. Fifth, potential confounders that could affect body composition and decrease bone mass, such as calcium intake and renal insufficiency, were not available in this study.

## Conclusion

In conclusion, fat mass, but not muscle mass, is a significant determinant of SI in community-dwelling postmenopausal Japanese women. This study suggests that body fat may be more important than muscle in maintaining good bone health in postmenopausal women.

## Data Availability

The datasets are available from the corresponding author for reasonable request. The datasets of the Unzen study analyzed in the current study are not publicly available because they include in-depth information, and we are planning to report other association studies using the same dataset.

## Abbreviations

DXA: Dual x-ray absorptiometry
BMD: Bone mineral density
SI: Stiffness index
QUS: Quantitative ultrasound
BIA: Bioelectrical impedance analysis
25(OH)D: 25-Hydroxyvitamin D
TRACP-5b: Tartrate-resistant acid phosphatase-5b
PTH: Parathyroid hormone
BUA: Broadband ultrasound attenuation
SOS: Speed of sound
BMI: Body mass index
CLEIA: Chemiluminescence enzyme immunoassay
ECLIA: Electrochemiluminescence immunoassay
SD: Standard deviation
SE: Standard error
